# Supplementary material for: Historically inconsistent productivity and respiration fluxes in the global terrestrial carbon cycle
Source: Nat Commun. 2022 Apr 1;13:1733. doi: 10.1038/s41467-022-29391-5 (PMC8976082; doi:10.1038/s41467-022-29391-5)
Supplement: Supplementary file 1 — Supplementary Information [file 41467_2022_29391_MOESM1_ESM.pdf]

Supplemental information for:

Historically inconsistent productivity and respiration fluxes in the global terrestrial carbon cycle

Authors: Jinshi Jian[1,2,3,4*], Vanessa Bailey[5], Kalyn Dorheim[2], Alexandra G. Konings[6], Dalei Hao[7], Alexey N. Shiklomanov[8], Abigail Snyder[2], Meredith Steele[9], Munemasa Teramoto[10,12], Rodrigo Vargas[11], and Ben Bond-Lamberty[2]

Affiliations:

1. State Key Laboratory of Soil Erosion and Dryland Farming on the Loess Plateau, Northwest A&F University, Yangling, 712100, China
2. Pacific Northwest National Laboratory, Joint Global Change Research Institute at the University of Maryland–College Park, 5825 University Research Court, Suite 3500, College Park, MD 20740, USA
3. University of Chinese Academy of Sciences, Beijing 100049, China
4. Institute of Soil and Water Conservation, Northwest A & F University, Yangling, Shaanxi 712100, China
5. Biological Sciences Division, Pacific Northwest National Laboratory, Richland, WA, 99354, USA
6. Department of Earth System Science, Stanford University, 473 Via Ortega, Room 140, Stanford, CA, 94305, USA
7. Atmospheric Sciences and Global Change Division, Pacific Northwest National Laboratory, Richland, WA, 99354, USA
8. NASA Goddard Space Flight Center, 8800 Greenbelt Rd., Building 33, Greenbelt, MD 20771, USA
9. School of Plant and Environmental Sciences, Virginia Tech, 183 Aq Quad Ln, Blacksburg, VA, 24061, USA
10. National Institute for Environmental Studies, 16-2 Onogawa, Tsukuba 305-8506, Japan
11. Present address: Arid Land Research Center, Tottori University, 1390 Hamasaka, Tottori 680–0001, Japan
12. Department of Plant and Soil Sciences, University of Delaware, Newark, DE, 19716 USA

* Corresponding author. Email: jinshi@vt.edu

Important acronyms: GPP_{Rs} – GPP estimated from R_S, GPP_{lit} – GPP reported from literature, Rs_{GPP} – R_S estimated from GPP, Rs_{lit} – R_S reported from literature.

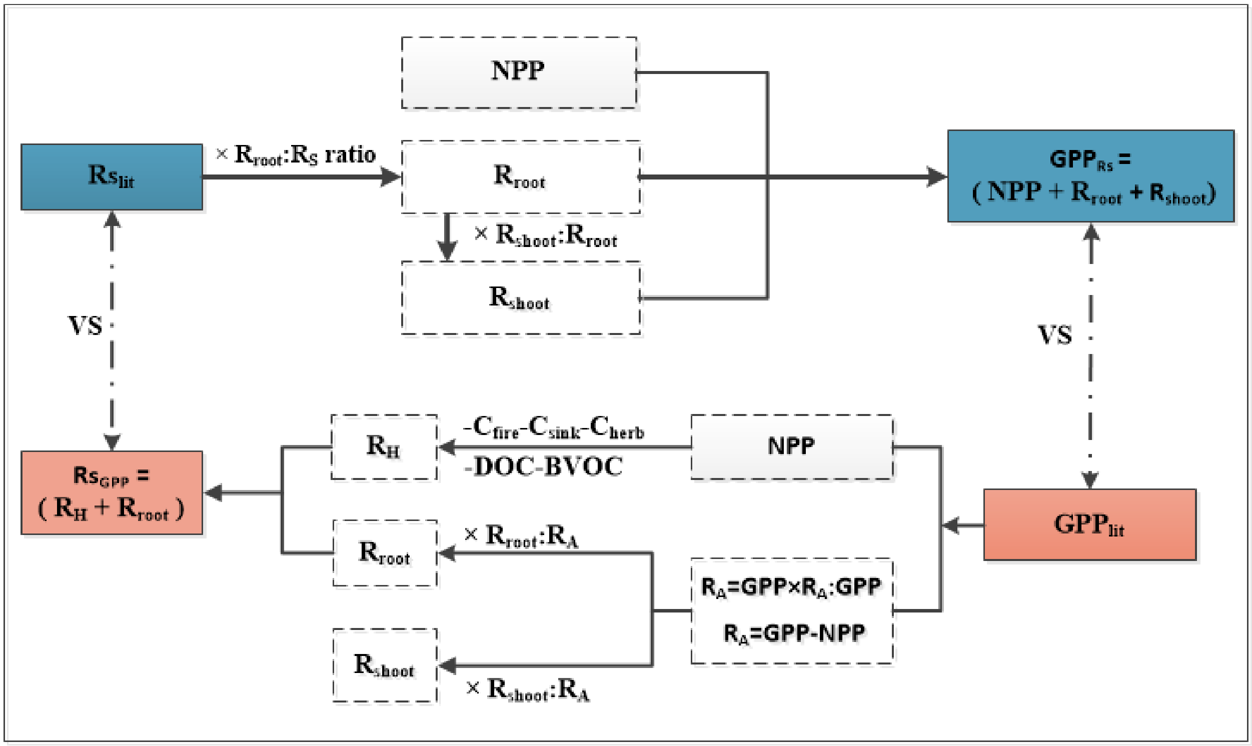

Figure 1. Summary diagram showing the computational chain used to infer gross primary productivity (GPP_{Rs}) from published global soil respiration (Rs_{lit}) and vice-versa. All units are Pg C yr^{-1}. Abbreviations used include NPP (net primary production), R_A (autotrophic respiration), R_{root} (root respiration), R_{shoot} (shoot respiration), R_H (heterotrophic respiration), C_{fire} (carbon components burned by fire), C_{sink} (the terrestrial carbon sink), C_{herb} (carbon consumed by herbivores), DOC (dissolved organic carbon), BVOC (biogenic volatile organic compound emissions), GPP_{lit} (global GPP estimates from literature), and Rs_{GPP} (global R_S implied from GPP_{lit}). See supplementary Figures 2-7 and supplementary Tables 1-5 for details and references.

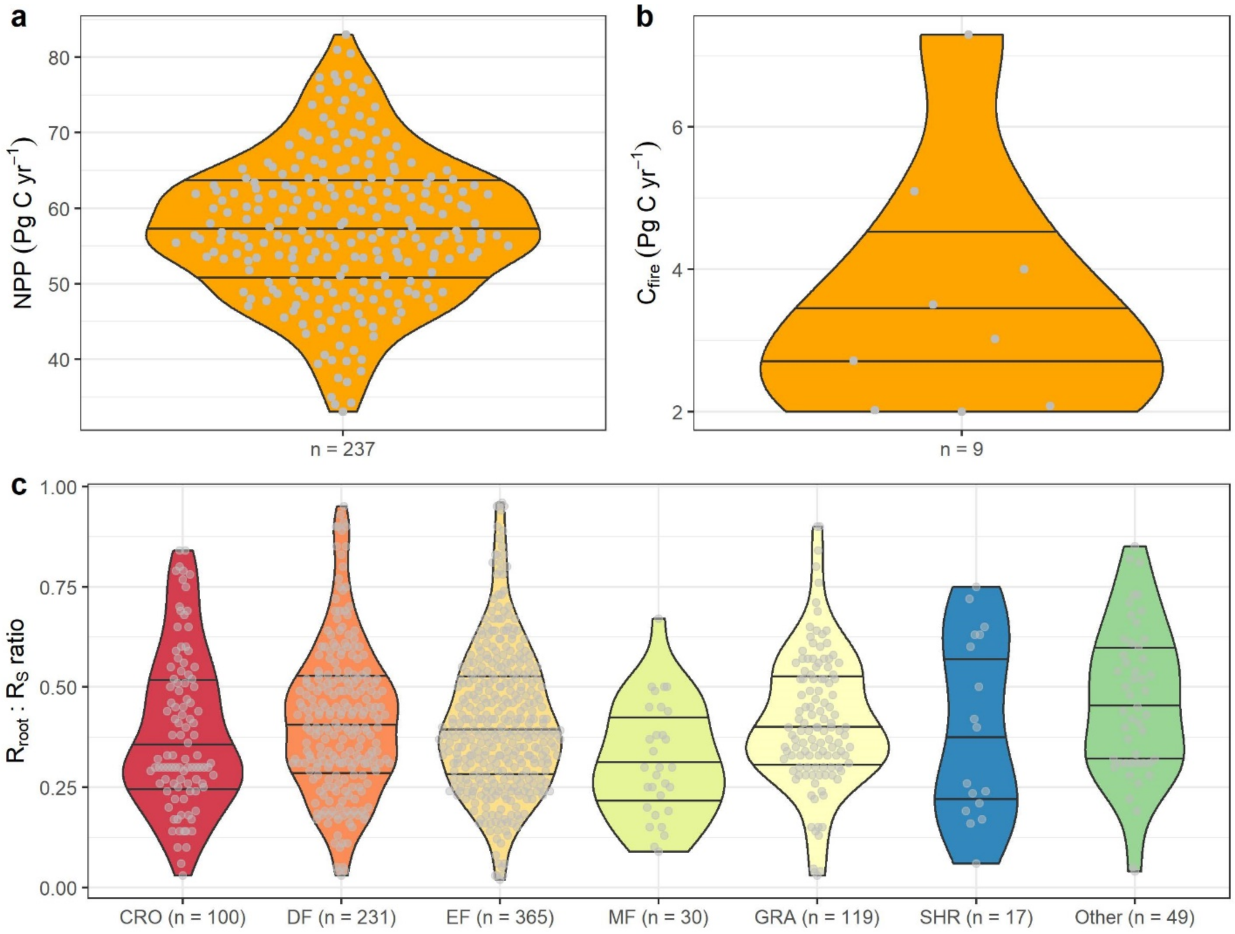

Figure 2. Net primary production (NPP), fire burned carbon (C_{fire}), and ratio of root respiration to total soil respiration (R_{root}:R_S) collected from the literature. (a) distribution of NPP collected from the literature; (b) distribution of C_{fire} collected from the literature; (c) distribution of R_{root}:R_S ratio reported from the literature, R_{root}:R_S ratio were grouped into cropland (CRO), deciduous forest (DF), evergreen forest (EF), mixed forest (MF), grassland (GRA), shrubland (SHR), and other vegetation types (i.e., desert, wetland, and savanna). Violin plots (enclosed areas) show distribution of each group; inside lines show the 25%, 50%, and 75% quantiles in each distribution.

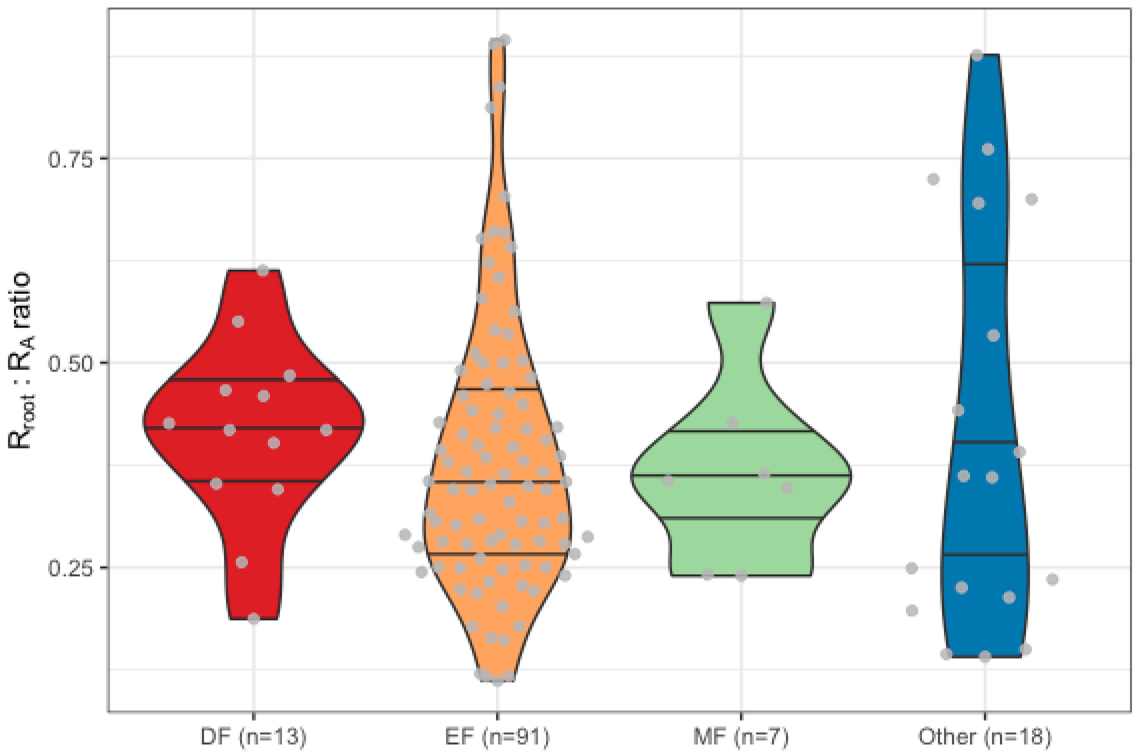

Figure 3. Ratio of root respiration to total autotrophic respiration (R_{root}:R_A) grouped by vegetation types. Violin plots (enclosed areas) show distribution of R_{root}:R_A. Inside lines show the 25%, 50%, and 75% quantiles in each distribution. The majority of data are from deciduous forests (DF), evergreen forests (EF), and mixed forests (MF); other vegetation types (cropland, savanna, grassland, and wetland) have only 18 observations combined.

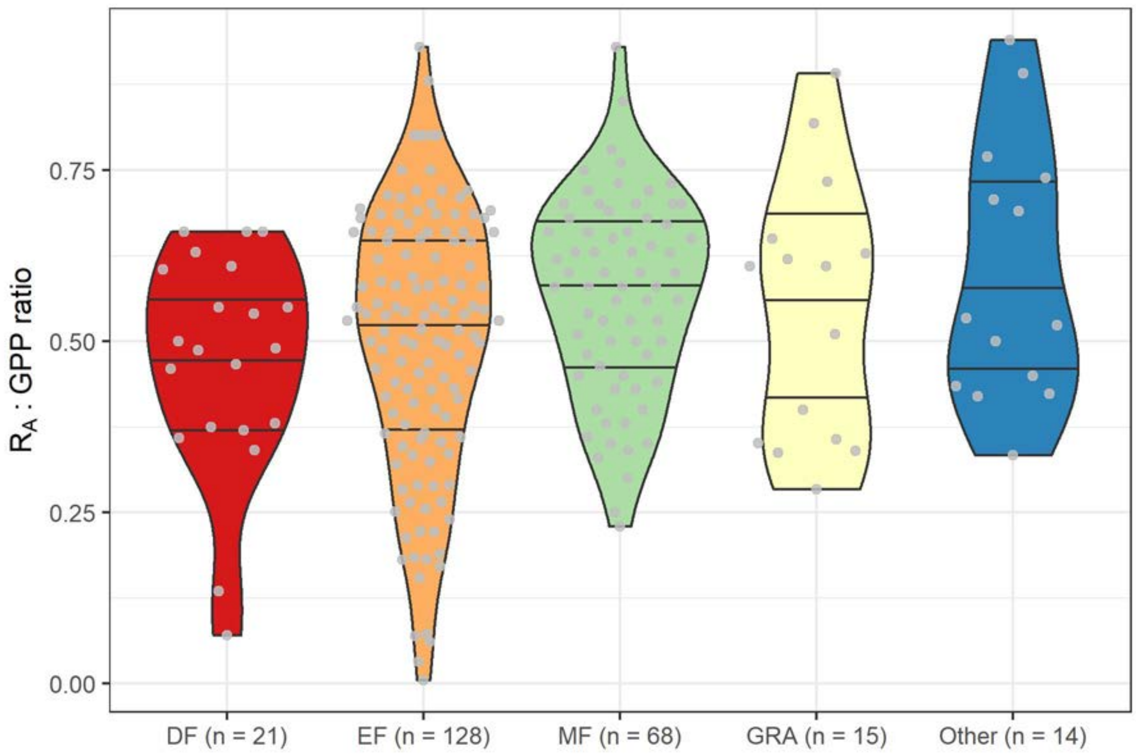

Figure 4. Ratio of total autotrophic respiration to gross primary productivity (R_A:GPP) by vegetation type. Violin plots (enclosed areas) show distribution by vegetation. Interior lines show the 25%, 50%, and 75% quantiles in each distribution. Vegetation types include deciduous forest (DF), evergreen forest (EF), mixed forest (MF), grassland (GRA), and other (including cropland, wetland, and tundra).

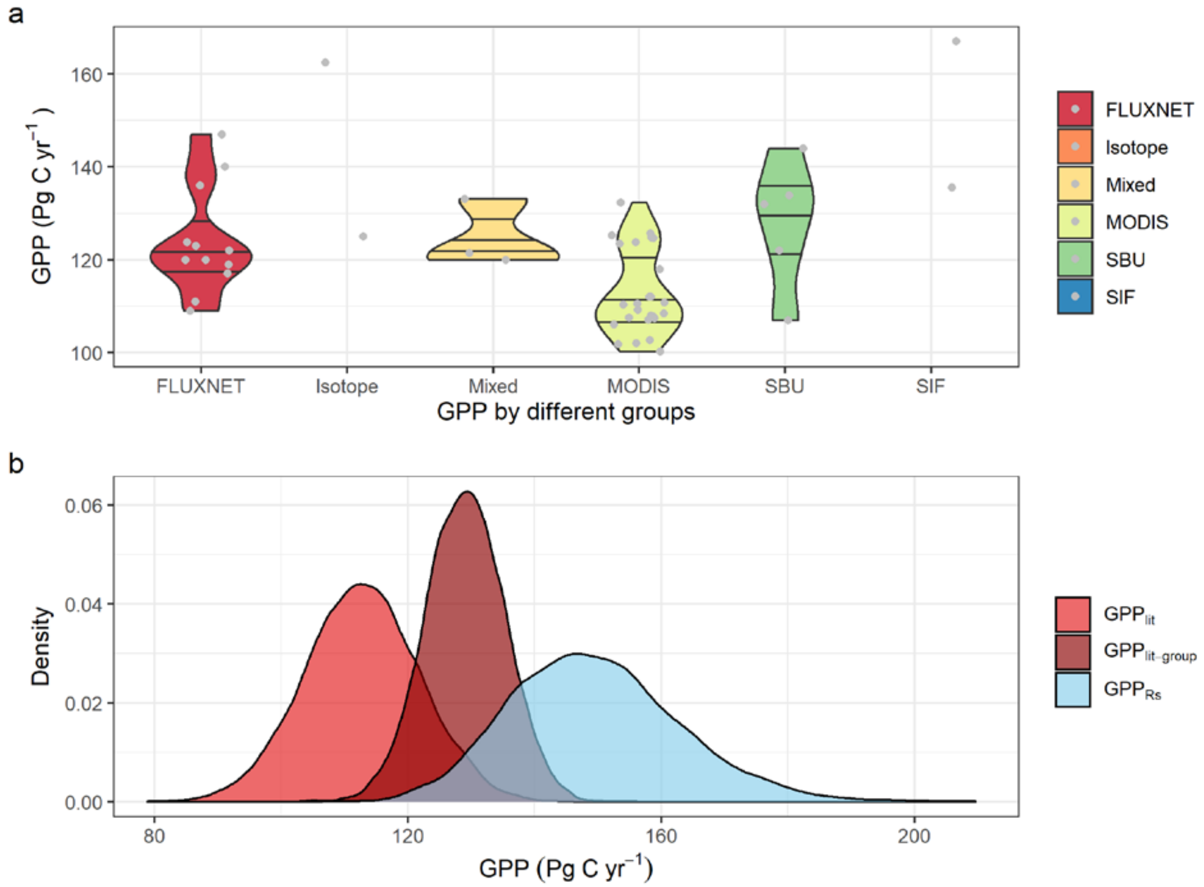

Figure 5. Distribution of global gross primary productivity (GPP) reported from the literature. (a) Violin plots of GPP estimated by different methods; (b) distributions of global GPP estimates bootstrapped from the raw data (GPP_{lit}) or aggregated by GPP groups before bootstrap resampling ($GPP_{lit-group}$) compared with the GPP implied by soil respiration (GPP_{Rs}). FLUXNET (GPP estimated based on FLUXNET sites data and upscaling approaches), Isotope (GPP estimated based on atmospheric isotope data (i.e., ^{18}O and ^{13}C, ref 1 and 2, respectively)), Mixed (GPP estimated by mixing satellite and site measurements), MODIS (GPP results using models driven by MODIS remote sensing images), SBU (*in-situ* based upscaling approaches), SIF (GPP estimated based on solar-induced chlorophyll fluorescence).

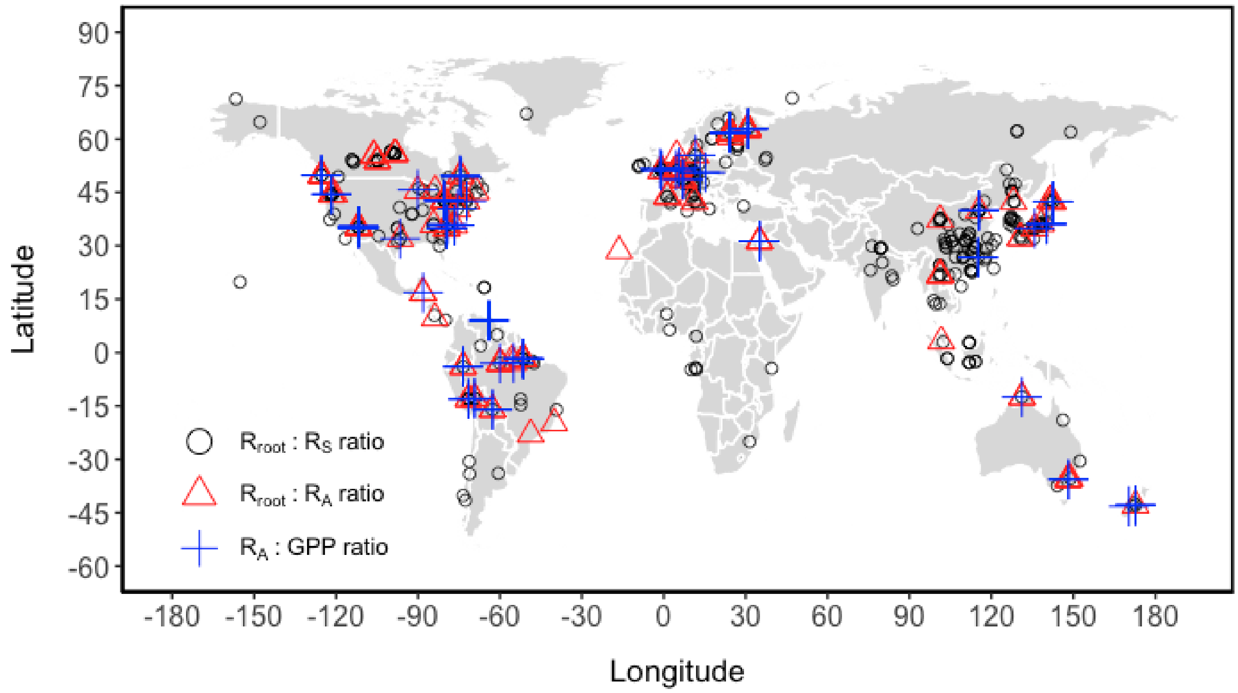

Figure 6. Spatial distribution of site data sources for the ratios of root respiration to total soil respiration (R_{root}:R_S), root respiration to total autotrophic respiration (R_{root}:R_A), and autotrophic respiration to gross primary productivity (R_A:GPP) used in this study. The R_{root}:R_S, R_{root}:R_A, and R_A:GPP sites have similar spatial coverage.

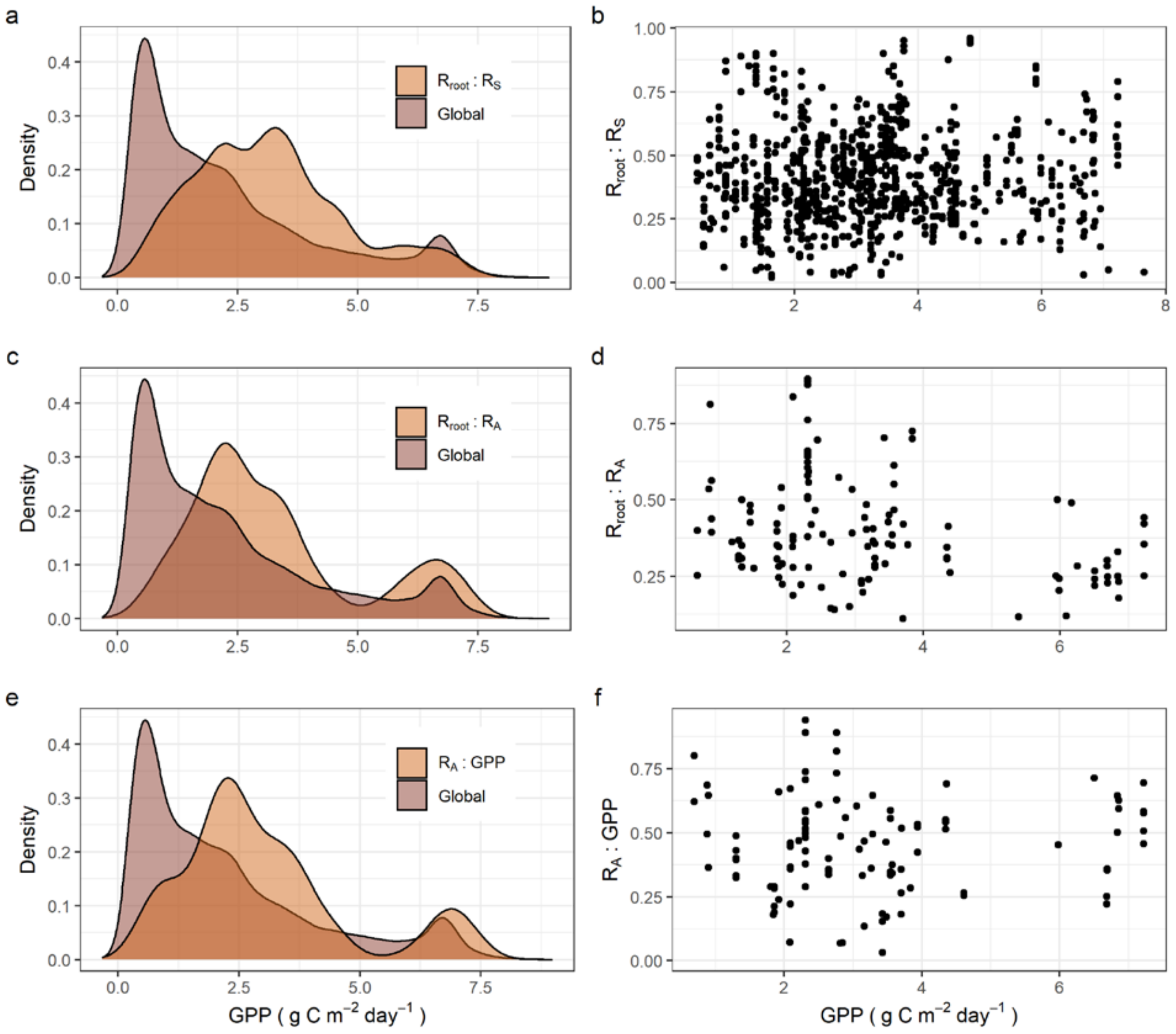

Figure 7. Comparison of gross primary productivity (GPP) coverage of ratio sites vs. global GPP from FLUXCOM. (a) Distribution of GPP coverage of R_{root}:R_S ratio sites used in this study compared with the distribution of global GPP from FLUXCOM; (b) scatter plot between R_{root}:R_S and GPP from sites used in this study; (c) distribution of GPP coverage of R_{root}:R_A ratio sites used in this study compared with the distribution of global GPP from FLUXCOM; (d) scatter plot between R_{root}:R_A ratio and GPP from sites used in this study; (e) distribution of GPP coverage of R_A:GPP ratio sites used in this study compared with the distribution of global GPP from FLUXCOM; (f) scatter plot between R_A:GPP ratio and GPP from sites used in this study. Generally, the ratio data used in this study have a similar coverage compared with the global GPP, but lack coverage of environments with low production. GPP data are obtained from FLUXCOM [3], averaged between 2001-2015 and scaled to 0.5° spatial resolution. FLUXCOM data are downloaded from https://www.fluxcom.org/ (last access date: 2021/06/22).

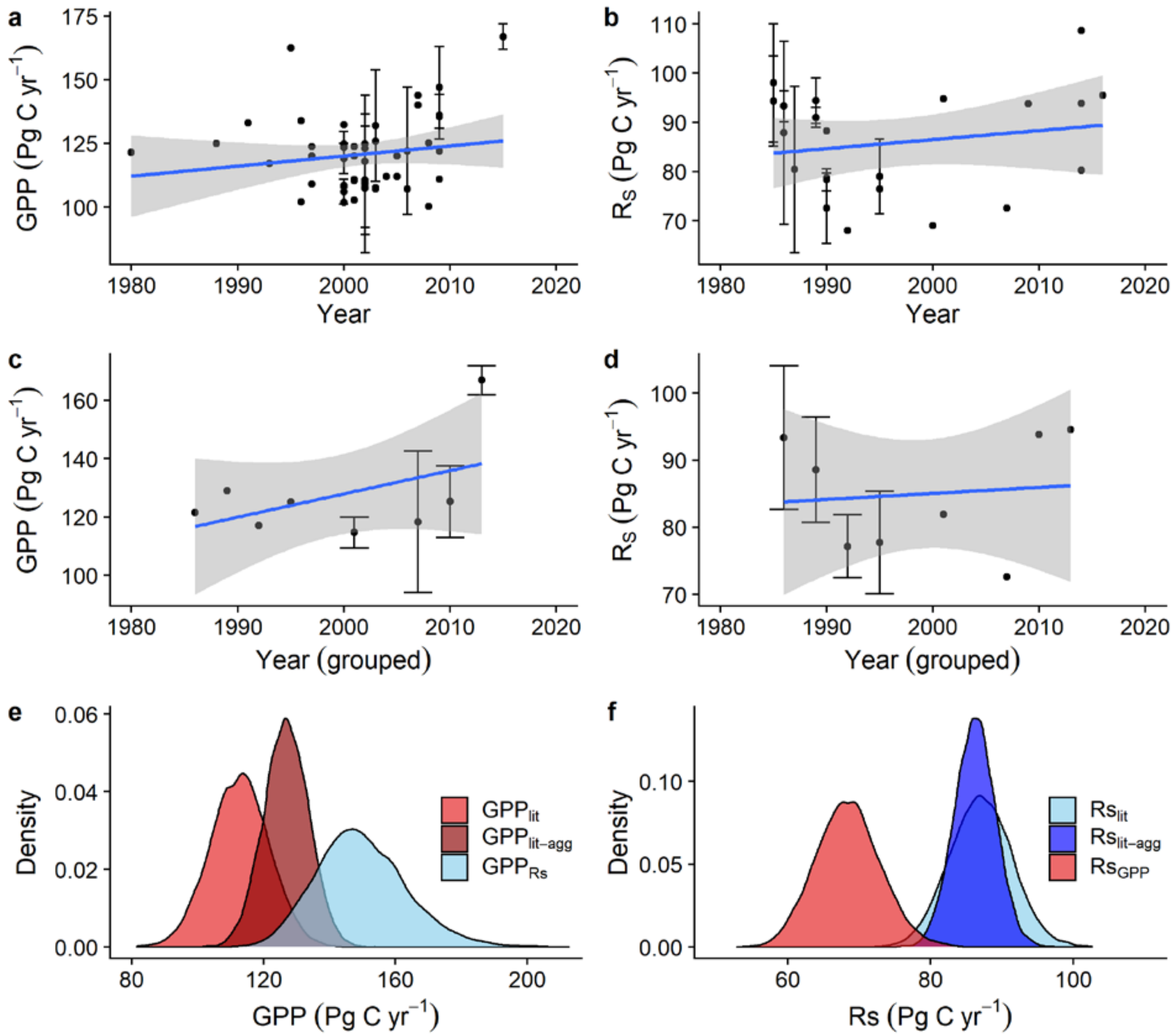

Figure 8. Pattern and distribution of global annual gross primary productivity (GPP) and soil respiration (R_S) collected from the literature. (a) and (b) show the trend of global R_S and GPP between 1980 and 2017, respectivily; (c) and (d) show the trend of aggregated global R_S and GPP between 1980 and 2017, respectively; (e) distribution of global GPP estimates bootstrapped from the raw data (GPP_{lit}) or aggregated by year before bootstrap resampling ($GPP_{lit\text{-}agg}$) compared with the GPP implied by R_S (GPP_{Rs}); (f) distribution of global R_S estimates bootstrapped from the raw data (Rs_{lit}) or aggregated by year before bootstrap resampling ($Rs_{lit\text{-}agg}$) compared with the R_S implied by GPP (GPP_{Rs}). For more details about the R_S and GPP data see supplementary Tables 1 and 2. Note that error bars are standard deviation reported in the literature.

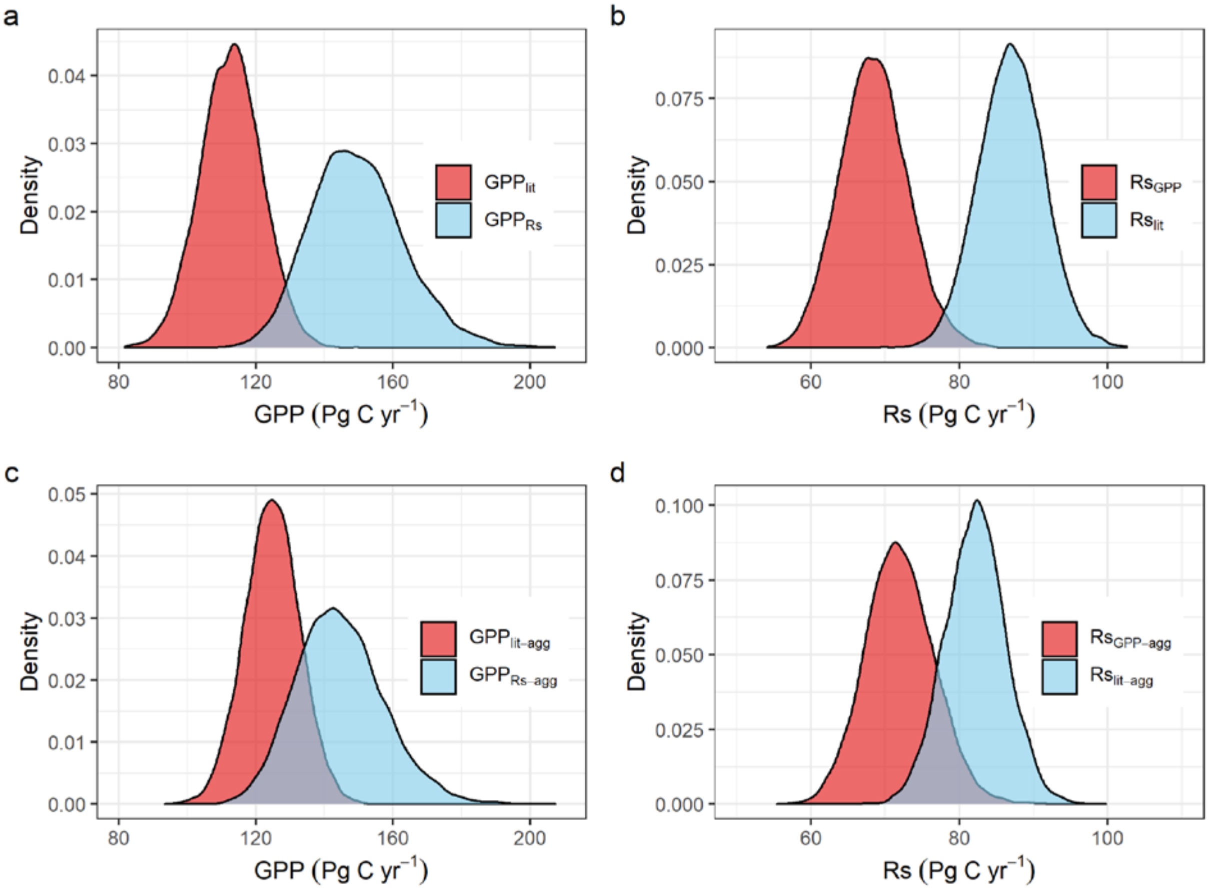

Figure 9. Distributions of global gross primary productivity (GPP) and global soil respiration (R_S) estimates bootstrapped from the raw data (a and b) or aggregated by year and GPP groups before bootstrap resampling (c and d). GPP_{lit} (GPP collected from the literature), $GPP_{lit-agg}$ (GPP aggregated by method groups and year before bootstrap resampling), Rs_{lit} (R_S collected from the literature), $Rs_{lit-agg}$ (R_S aggregated by year before bootstrap resampling). Distributions are based on 10,000 random draws of the underlying estimates from published literature (summarized in supplementary Tables 1 and 2).

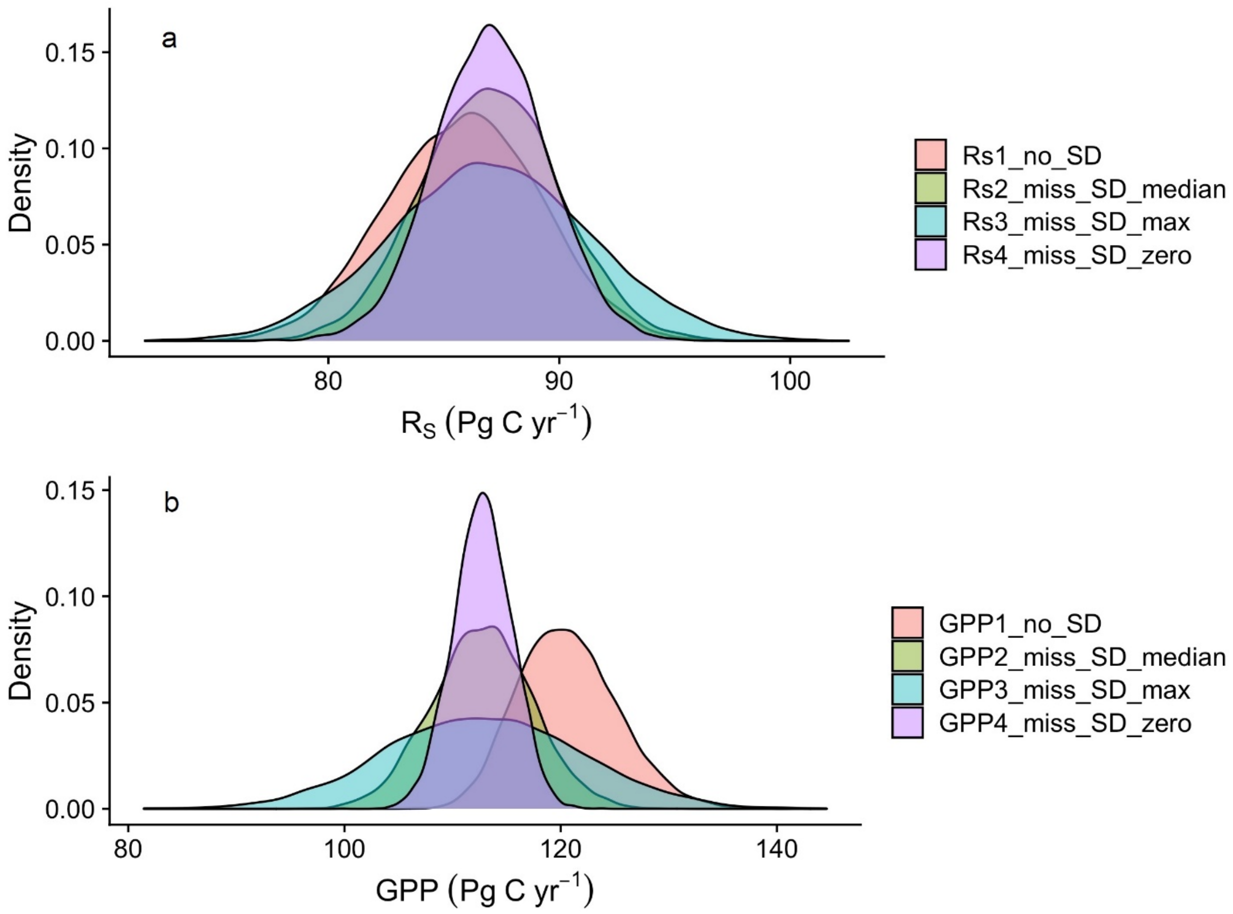

Figure 10. Comparison of four different methods (each N = 10,000 with replacement) for resampling global soil respiration (R_S; a) and global primary productivity (GPP; b) estimates. Method 1 does not use error information when resampling. Methods 2-4 use errors, but handle missing values differently: method 2 replaces missing errors with values calculated from the median coefficient of variability (CV) of non-missing values; method 3 replaces missing errors with values calculated from the maximum CV across the dataset; and method 4 sets missing errors to zero. We used method 3 in the main analysis, which is the most conservative (produces the widest distribution for both R_S and GPP).

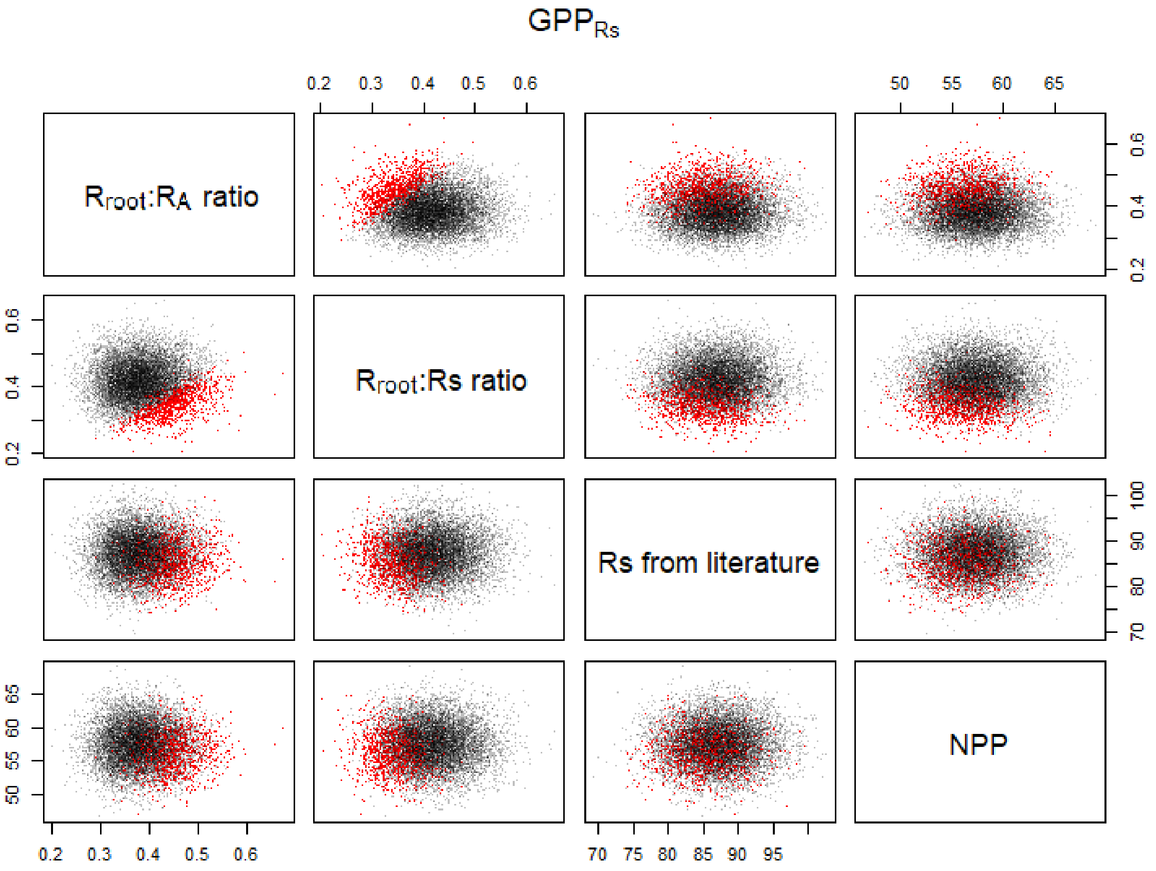

Figure 11. Relationship between calculated GPP_{Rs} (global gross primary productivity as driven by global soil respiration (Rs) flux estimates) and the various partitioning variables, all defined in Table 1 in the main text. Black dots are those for which GPP_{Rs} was below the intersection point (127.6 Pg C yr^{-1}), while the red dots are above the intersection in Figure 1a in the main text. R_{root} (root respiration), R_A (autotrophic respiration); NPP (net primary productivity).

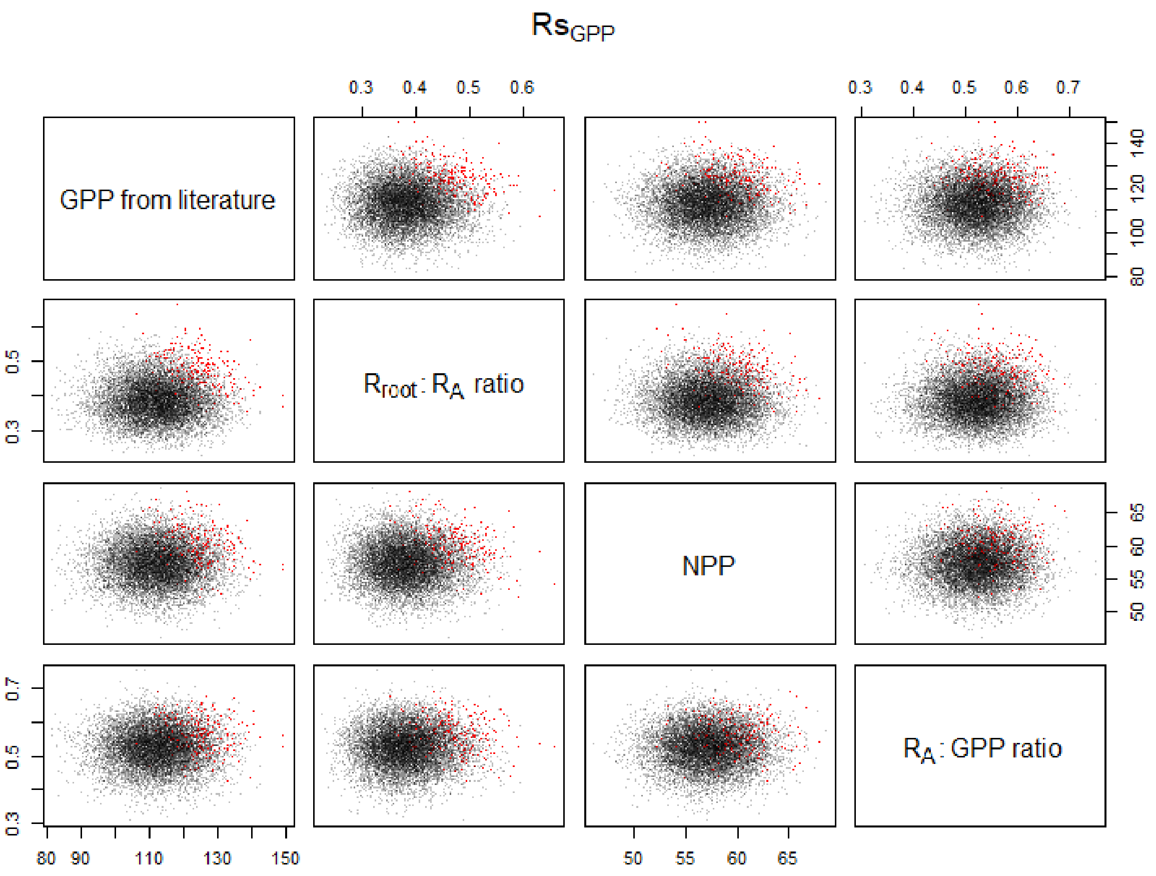

Figure 12. Relationship between Rs_{GPP} (global soil respiration as driven by gross primary productivity (GPP) estimates from the literature) and the partitioning variables, as defined in Table 1 in the main text. Black dots are those Rs_{GPP} below the intersection point (78.2 Pg C yr^{-1}) in Figure 1b in the main text, while the red dots are above it. R_{root} (root respiration), R_A (autotrophic respiration).

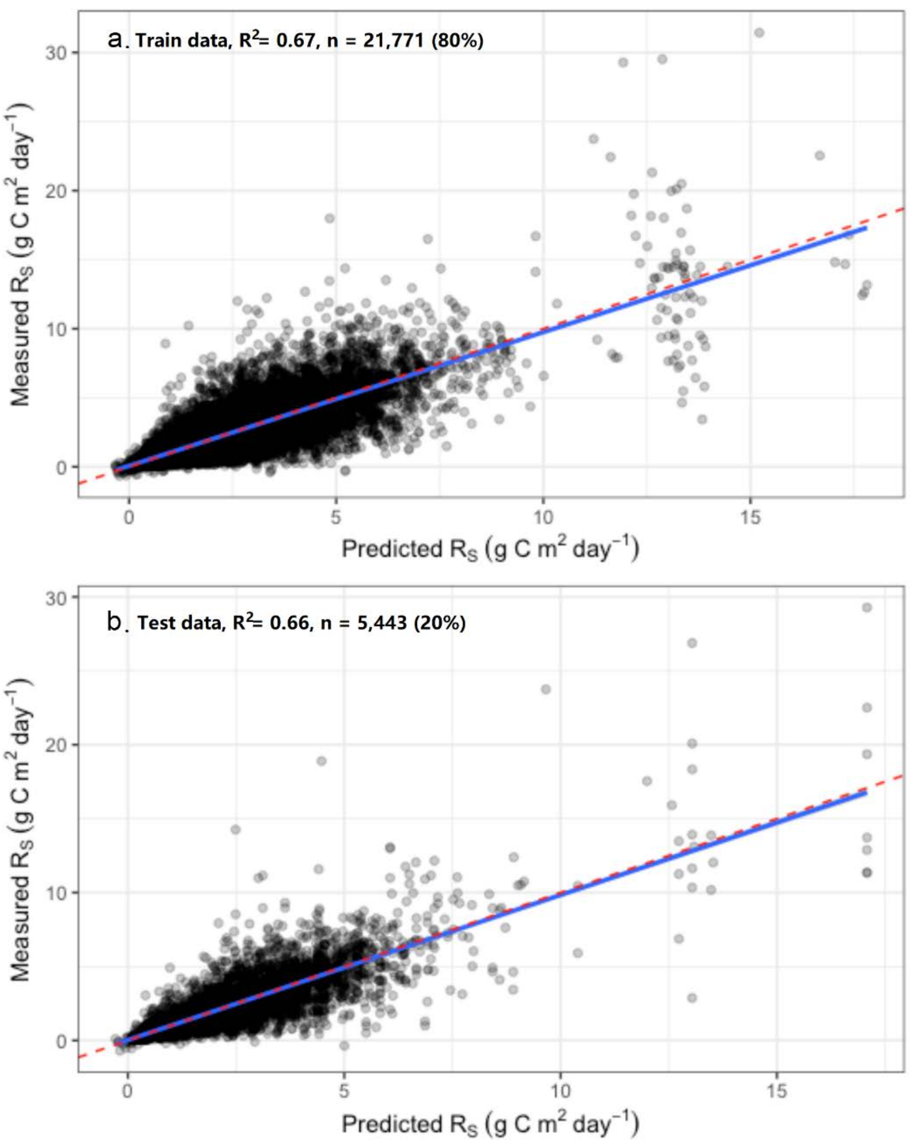

Figure 13. Relationship between measured soil respiration (R_S) and predicted R_S using Random Forest modeling. We used 80% samples to train the Random Forest model (a), the rest of R_S samples (20%) were used to test the model performance (b). The results showed that the Random Forest model explains approximately 66% R_S variability, and the performance is very consistent between the train and test dataset. The regression line between predicted and measured R_S (solid line) is very close to the 1:1 dashed line.

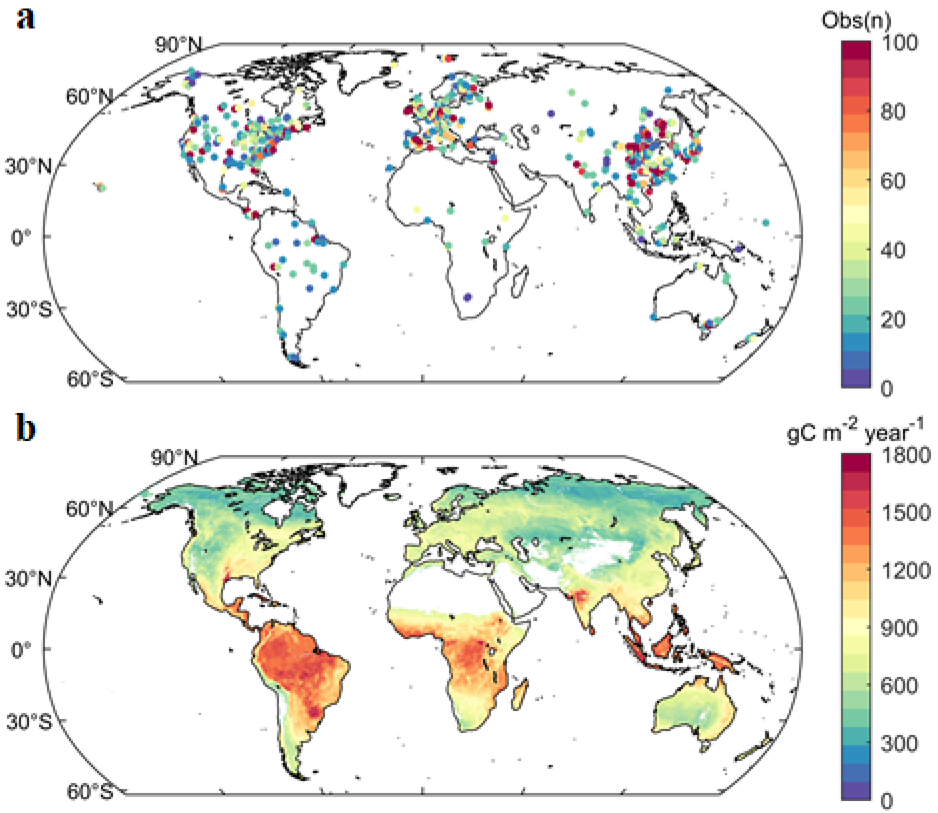

Figure 14. Spatial distribution of soil respiration (R_S) sites and predicted global R_S. (a) Spatial distribution of sites used in the Random Forest model; (b) Global spatial distribution of R_S predicted by the Random Forest model, with spatial resolution of 0.1° latitude × 0.1° longitude. We used the International Geosphere-Biosphere Programme (IGBP) classification layer in the MCD12Q1 to mask all pixels with the land cover types of snow/ice, water, and barren.

Table 1. Global soil respiration estimates from the literature (Rs_{lit}, Pg C yr^{-1}), with any reported 95% confidence interval or stadard deviation (CI or SD, Pg C yr^{-1}) and trend (Pg C yr^{-2}). Note that n.a. means not available; [a] Confidence interval; [b] Standard deviation; [†]from ref [9].

Year	Period	Rs_{lit}	CI or SD	Trend	Ref.
1989	1966-2012	91.0	4.0[a]	0.09	[4]
1985	1962-2008	98.0	12.0[b]	0.10	[5]
1986	1961-2011	87.9	n.a.	n.a.	[6]
1990	1964-2016	78.8	n.a.	n.a.	[7]
1990	1964-2016	88.2	n.a.	n.a.	[7]
2014	n.a.	93.9	n.a.	n.a.	[8]
2014	n.a.	80.3	n.a.	n.a.	[8]
2014	n.a.	108.6	n.a.	n.a.	[8]
1985	1960-2010	94.3	17.9[a]	n.a.	[9]
1990	1964-2016	78.3	2.2[b]	0.03	[10]
1990	1964-2016	72.6	7.1[b]	0.03	[10]
1987	1980-1994	80.4	16.9[b, †]	0.10	[11]
1977	n.a.	75.0	n.a.	n.a.	[12]
1992	n.a.	68.0	4.0[b]	n.a.	[13]
1995	n.a.	76.5	n.a.	n.a.	[14]
1995	1980-2010	79.0	15.5[a]	n.a.	[15]
1989	1970-2008	94.4	9.0[b]	0.04	[16]
2007	2000-2014	72.6	n.a.	0.13	[17]
1986	1960-2012	93.3	6.1[a]	0.04	[18]
2000	n.a.	69.0	n.a.	n.a.	[19]
2016	n.a.	95.5	n.a.	n.a.	[20]
2001	n.a.	94.8	n.a.	n.a.	21
2009	n.a.	93.8	n.a.	n.a.	21

Table 2. Global gross primary productivity collected from the literature (GPP_{lit}, Pg C yr^{-1}), with any accompanying standard deviation (SD, Pg C yr^{-1}) and trend (Pg C yr^{-2}). Note that n.a. means not available.

Year	Period	GPP_{lit}	SD	Trend	Notes	Ref.
2002	2001-2003	109.3	27.3	n.a.	MODIS	[22]
2015	n.a.	167.0	5.0	n.a.	SIF	[23]
2009	2000-2017	135.5	8.8	n.a.	SIF	[24]
2004	1997-2010	112.0	n.a.	0.01	MODIS	[25]
1991	n.a.	133.1	n.a.	n.a.	TURC-Satellite	[26]
2009	2008-2010	111.0	n.a.	n.a.	FLUXCOM	[3]
2009	2008-2010	122.0	n.a.	n.a.	FLUXCOM	[3]
2009	2008-2010	136.0	n.a.	n.a.	FLUXCOM	[3]
2005	2000-2010	112.0	n.a.	0.28	MODIS	[27]
2005	2000-2010	120.0	n.a.	n.a.	FLUXNET-MTE	[27]
1995	1980-2009	162.5	n.a.	n.a.	O^{18}	[1]
2000	2000-2003	108.0	n.a.	n.a.	MODIS	[28]
2001	2000-2003	110.3	n.a.	n.a.	MODIS	[28]
2002	2000-2003	107.4	n.a.	n.a.	MODIS	[28]
2003	2000-2003	107.1	n.a.	n.a.	MODIS	[28]
2001	n.a.	120.0	n.a.	n.a.	IPCC	[29]
2006	2000-2011	107.0	n.a.	n.a.	Satellite	[30]
2002	2001-2003	118.0	26.0	n.a.	MODIS	[31]
1993	1982-2004	117.0	n.a.	n.a.	FLUXNET-MTE	[32]
2002	1998-2005	123.0	n.a.	n.a.	FLUXNET-MTE	[33]
2000	1992-2008	119.0	6.0	0.08	FLUXNET-MTE	[34]
1988	1981-1994	125.0	n.a.	n.a.	Isotope	[2]
2009	2003-2015	147.0	16.0	n.a.	NIRv	[35]
2007	n.a.	140.0	n.a.	n.a.	SIF	[36]

2002	2000-2003	110.5	21.3	n.a.	MODIS	[37]
2008	2000-2016	125.2	n.a.	0.39	MODIS-VPM	[38]
2006	2001-2011	122.0	25.0	0.27	BESS	[39]
2000	1985-2015	124.7	5.0	0.45	MODIS MOD17A2H	[40]
2000	1985-2015	106.0	5.0	0.45	MODIS MOD17A2H	[40]
2003	2003	132.0	22	n.a.	BEPS	[41]
1980	1953-1999	121.5	n.a.	n.a.	MODIS	[42]
2000	2001-2003	108.4	n.a.	n.a.	MODIS	[43]
2001	2001-2003	110.8	n.a.	n.a.	MODIS	[43]
2002	2001-2003	107.8	n.a.	n.a.	MODIS	[43]
2003	2001-2003	107.5	n.a.	n.a.	MODIS	[43]
2000	2001-2003	101.8	n.a.	n.a.	MODIS	[43]
2001	2001-2003	102.7	n.a.	n.a.	MODIS	[43]
2002	2001-2003	124.8	n.a.	n.a.	MODIS	[43]
2003	2001-2003	125.8	n.a.	n.a.	MODIS	[43]
2000	2001-2003	123.4	n.a.	n.a.	MODIS	[43]
2001	2001-2003	123.7	n.a.	n.a.	MODIS	[43]
2000	2000-2001	132.3	n.a.	n.a.	MODIS	[44]
2007	2000-2014	144.0	n.a.	0.07	FLUXNET-SVR	[45]
1997	1982-2013	109.0	n.a.	0.01	FLUXCOM-ANN	[45]
1997	1982-2013	120.0	n.a.	-0.01	FLUXCOM-MARS	[45]
1997	1982-2013	123.8	n.a.	0.02	FLUXCOM-RF	[45]
2008	2001-2016	100.2	n.a.	0.36	MODIS-C6	[45]
1996	1982-2011	133.9	n.a.	0.30	Global carbon data (PR)	[45]
1996	1982-2011	102.0	n.a.	0.14	MODIS-GIMMS	[45]

Table 3. Summary of potential biases when using *in-situ* soil respiration (R_S) measurements to estimate global R_S and when using remote sensing technology to estimate global GPP.

**Bias**	**Description and method to reduce related bias**
Estimate global R_S based on field measurements	
Measurement protocols and R_S temporal variability	• More than 97% R_S measurements in SRDB involve collar insertion, which likely lead to root mortality and reduced R_S [46].<br>• *In situ* Rs measurements may not be representative of Rs at ecosystem-scale [47].<br>• Global R_S estimated by the RF model parameterized based on the monthly global R_S database (79 Pg) is about 9 Pg smaller than global R_S estimated by the RF model parameterized based on the annual global R_S database (88 Pg) [7].<br>• A new study showed that upto 2/3 of R_S was removed by subsurface processes, therfore, measuring vertical (upward) gaseous may significantly underestimate R_S [48].
R_S measurement frequency and measurement time	Overall, measurement time and frequency causes no significant bias on annual R_S, possibly due to canceling effects [46].
Estimate global GPP based on remote sensing technology	
Remote sensing image related	• Remote sensing signals become less reliable over time due to sensor degradation [49,50].<br>• Cloud contamination [51–53].<br>• In areas with sparse vegetation, soil background albedo influences reflectivity [54].
Eddy covariance related	• Products such as FLUXCOM do not account for all C loss pathways or CO_2 fertilization effects [3].<br>• Uncertainties and mismatches in the algorithms that partition towers' net ecosystem exchange into GPP and respiration [55].

Table 4. Summary of global carbon fluxes from published literature. Net primary production (NPP), herbivore consumption (C_{herb}), fire losses (C_{fire}), dissolved organic carbon (DOC) exports, biogenic volatile organic compound emissions (BVOC), and terrestrial carbon sink to terrestrial ecosystem (C_{sink}). Note that n.a. means not available.

**Flux**	**Period**	**Amount (Pg C yr^{-1})**	**Ref.**
NPP	1862-2011	56.20 (average of 237 observations)	[56]
C_{herb}	n.a.	1.40 (± 0.20)	[57]
	n.a.	3.00	[58]
C_{fire}	1997-2016	2.20	[59]
	1960s	3.50 (± 1.50)	[60]
	n.a.	7.30	[61]
	1901-2002	4.00	[62]
	1980-2000	5.10	[63]
	1920-1970	2.02	[64]
	1970-2010	2.71	[64]
	1900-2000	3.02 (± 0.30)	[65]
	1960-2000	2.08	[66]
C_{sink}	1959-2014	2.10 (± 0.28)	[67]
DOC	n.a.	1.90	[68]
	n.a.	2.90	[69]
	n.a.	2.10 (±0.25)	[70,71]
BVOC	n.a.	1.007	[72]

Table 5. Summary of field measured root respiration to autotrophic respiration ratio (R_{root}:R_A) collected from studies.

Year	R_{root}:R_A (%)	R_{shoot}:R_A (%)	Vegetation type	Ref.	Temporal coverage
1997	71.00	29.00	EF	[73]	Annual
1997	77.70	22.30	EF	[73]	Annual
1997	53.98	46.02	EF	[73]	Annual
1997	51.77	48.23	EF	[73]	Annual
1997	57.35	42.65	DF	[73]	Annual
1997	75.54	24.45	EF	[73]	Annual
1997	71.78	28.22	EF	[73]	Annual
1997	65.42	34.58	DF	[73]	Annual
1976	50.00	50.00	EF	[74]	Annual
1983	88.00	12.00	EF	[75]	Annual
1978	55	45.00	EF	[76]	Annual
1981	54.00	46.00	DF	[77]	Annual
1996	72.50	27.5	EF	[78]	Annual
1999	46.52	53.48[†]	EF	[79]	Annual
2011	75.08	24.92	CRO	[80]	Growing season
2000	53.30	46.70	DF	[81]	Annual
2003	30.00	70.00	SAV	[82]	Annual
2004	42.68	57.32[†]	MF	[83]	Annual
2005	57.28	42.72	MF	[84]	Annual
2006	70.99	29.01	EF	[85]	Annual
2006	65.22	34.78	MF	[86]	Annual
2006	50.96	49.04	EF	[87]	Annual
2007	63.57	36.43[†]	EF	[88]	Annual
2007	63.29	36.71[†]	EF	[89]	Annual

2009	64.43	35.57	EF	[90]	Annual
2009	53.53	46.47†	EF	[91]	Annual
2009	71.72	28.28	EF	[92]	Annual
2009	50.01	49.99	EF	[93]	Annual
2009	63.79	36.21	GRA	[94]	July
2010	65.45	34.55	EF	[95]	Annual
2010	57.27	42.73†	EF	[96]	Annual
2010	74.94	25.06	EF	[97]	Annual
2010	76.45	23.55	CRO	[98]	Annual
2012	73.85	26.15†	EF	[99]	Annual
2015	76.00	24.00†	MF	[100]	Annual

† Root respiration estimated based on soil respiration from ref. [101].

Table 6. Global factors used for monthly soil respiration (R_S) random forest modeling and predicting the global R_S map. Note that n.a. means not available.

Group	Factors	Temporal averaged	Spatial resolution	Unit	Ref. and sources
Landcover	Landcover	2010	500m	Unitless	[102]
Climate	Monthly precipitation	1960-2018	1km	℃	[103]
	Monthly air temperature	1960-2018	1km	mm	
N nitrogen deposition	N-deposition	1980-2017	0.5° × 0.5°	g N m^{-2} yr^{-1}	[104] https://www.isimip.org/gettingstarted/details/24/
Soil	Clay content	n.a.	1km	Unitless	[105] https://files.isric.org/soilgrids/former/2017-03-10/data/ (last access: Dec. 2020)
	Bulk density	n.a.	1km	g cm^{-3}	
	SOC	n.a.	1km	Mg C ha^{-1}	
Biomass	Above ground biomass	2010	0.0028° × 0.0028°	Mg C ha^{-1}	[106] https://daac.ornl.gov/cgi-bin/dsviewer.pl?ds_id=1763
	Below ground biomass	2010	0.0028° × 0.0028°	Mg C ha^{-1}	
Enhanced Vegetation Index	EVI	2000-2017	1 km	Unitless	[107]

References for supplemental information

1. Welp, L. R. *et al.* Interannual variability in the oxygen isotopes of atmospheric CO2 driven by El Niño. *Nature* 477, 579–582, doi:10.1038/nature10421 (2011).
2. Randerson, J. T. *et al.* Carbon isotope discrimination of arctic and boreal biomes inferred from remote atmospheric measurements and a biosphere-atmosphere model. *Global Biogeochem. Cycles* 16, 1–15, https://doi.org/10.1029/2001GB001435 (2002).
3. Jung, M. *et al.* Scaling carbon fluxes from eddy covariance sites to globe: Synthesis and evaluation of the FLUXCOM approach. *Biogeoscices* 17, 1343–1365, https://doi.org/10.5194/bg-17-1343-2020 (2020).
4. Hashimoto, S. *et al.* Global spatiotemporal distribution of soil respiration modeled using a global database. *Biogeosciences* 12, 4121–4132, https://doi.org/10.5194/bg-12-4121-2015 (2015).
5. Bond-Lamberty, B. & Thomson, A. Temperature-associated increases in the global soil respiration record. *Nature* 464, 579–582, doi:10.1038/nature08930 (2010).
6. Warner, D. L., Bond-Lamberty, B., Jian, J., Stell, E. & Vargas, R. Spatial Predictions and Associated Uncertainty of Annual Soil Respiration at the Global Scale. *Global Biogeochem. Cycles* 33, 1733–1745, https://doi.org/10.1029/2019GB006264 (2019).
7. Jian, J., Steele, M. K., Thomas, R. Q., Day, S. D. & Hodges, S. C. Constraining estimates of global soil respiration by quantifying sources of variability. *Glob. Chang. Biol.* 24 4143-4159, https://doi.org/10.1111/gcb.14301 (2018).
8. Hursh, A. *et al.* The sensitivity of soil respiration to soil temperature, moisture, and carbon supply at the global scale. *Glob. Chang. Biol.* 23, 2090–2103, https://doi.org/10.1111/gcb.13489 (2017).
9. Xu, M. & Shang, H. Contribution of soil respiration to the global carbon equation. *J. Plant Physiol.* 203, 16–28, https://doi.org/10.1016/j.jplph.2016.08.007 (2016).
10. Jian, J., Steele, M. K., Day, S. D. & Thomas, R. Q. Future global soil respiration rates will swell despite regional decreases in temperature sensitivity caused by rising temperature. *Earth's Future* 6, 1539-1554, https://doi.org/10.1029/2018EF000937 (2018).
11. Raich, J. W., Potter, C. S. & Bhagawati, D. Interannual variability in global soil respiration, 1980-94. *Glob. Chang. Biol.* 8, 800–812, https://doi.org/10.1046/j.1365-2486.2002.00511.x (2002).

12. Schlesinger, W. H. Carbon balance in terrestrial detritus. *Annual Reviews in Ecology and Systematics* 8, 51–81, https://doi.org/10.1146/annurev.es.08.110177.000411 (1977).
13. Raich, J. W. & Schlesinger, W. H. The global carbon dioxide flux in soil respiration and its relationship to vegetation and climate. *Tellus B Chem. Phys. Meteorol.* 44, 81–99, https://doi.org/10.1034/j.1600-0889.1992.t01-1-00001.x (1992).
14. Raich, J. W. & Potter, C. S. Global patterns of carbon dioxide emissions from soils. *Global Biogeochem. Cycles* 9, 23–36, https://doi.org/10.1029/94GB02723 (1995).
15. Hashimoto, S. A new estimation of global soil greenhouse gas fluxes using a simple data-oriented model. *PLoS One* 7, e41962, https://doi.org/10.1371/journal.pone.0041962 (2012).
16. Chen, S. *et al.* A new estimate of global soil respiration from 1970 to 2008. *Chin. Sci. Bull.* 58, 4153–4160, doi: 10.1007/s11434-013-5912-1 (2013).
17. Huang, N. *et al.* Spatial and temporal variations in global soil respiration and their relationships with climate and land cover. *Sci. Adv.* 6, eabb8508, doi: 10.1126/sciadv.abb8508 (2020).
18. Zhao, Z., *et al*. Model prediction of biome-specific global soil respiration from 1960 to 2012. *Earth's Future.* 5, 715-729, https://doi.org/10.1002/2016EF000480 (2017).
19. Peng, C., and Apps, M. J. Simulating global soil-CO_2 flux and its response to climate change. *J. Environ. Sci*. 12, 257-265 (2000).
20. Oertel, C., Matschullat, J., Zurba, K., Zimmermann, F., & Erasmi, S. Greenhouse gas emissions from soils-A review. *Geochemistry* 76, 327-352, https://doi.org/10.1016/j.chemer.2016.04.002 (2016).
21. Adachi, M., Ito, A., Yonemura, S., & Takeuchi, W. Estimation of global soil respiration by accounting for land-use changes derived from remote sensing data. *J. Enviro. Manage.* 200, 97-104, https://doi.org/10.1016/j.jenvman.2017.05.076 (2017).
22. Zhao, M., Heinsch, F. A., Nemani, R. R. & Running, S. W. Improvements of the MODIS terrestrial gross and net primary production global data set. *Remote Sens. Environ.* 95, 164–176, https://doi.org/10.1016/j.rse.2004.12.011 (2005).
23. Norton, A. J. *et al.* Estimating global gross primary productivity using chlorophyll fluorescence and a data assimilation system with the BETHY-SCOPE model.

Biogeosciences 16, 3069–3093, https://doi.org/10.5194/bg-16-3069-2019 (2019).

24. Li, X. & Xiao, J. Mapping Photosynthesis Solely from Solar-Induced Chlorophyll Fluorescence: A Global, Fine-Resolution Dataset of Gross Primary Production Derived from OCO-2. *Remote Sensing* 11, 2563, https://doi.org/10.3390/rs11212563 (2019).
25. Anav, A. *et al.* Spatiotemporal patterns of terrestrial gross primary production: A review. *Rev. Geophys.* 53, 2015RG000483, https://doi.org/10.1002/2015RG000483 (2015).
26. Ruimy, A., Dedieu, G. & Saugier, B. TURC: A diagnostic model of continental gross primary productivity and net primary productivity. *Global Biogeochem. Cycles* 10, 269–285, https://doi.org/10.1029/96GB00349 (1996).
27. Chen, M. *et al.* Regional contribution to variability and trends of global gross primary productivity. *Environ. Res. Lett.* 12, 105005, https://doi.org/10.1088/1748-9326/aa8978 (2017).
28. Zhang, Y., Xu, M., Chen, H. & Adams, J. Global pattern of NPP to GPP ratio derived from MODIS data: effects of ecosystem type, geographical location and climate. *Glob. Ecol. Biogeogr.* 18, 280–290, https://doi.org/10.1111/j.1466-8238.2008.00442.x (2009).
29. Prentice, I. C. *et al.* The Carbon Cycle and Atmospheric CO_2. in *Contribution of Working Group I to the Fourth Assessment Report of the Intergovernmental Panel on Climate Change* (Cambridge University Press, Cambridge, 2001) 183–238.
30. Yebra, M., Van Dijk, A. I. J. M., Leuning, R. & Guerschman, J. P. Global vegetation gross primary production estimation using satellite-derived light-use efficiency and canopy conductance. *Remote Sens. Environ.* 163, 206–216, https://doi.org/10.1016/j.rse.2015.03.016 (2015).
31. Ryu, Y. *et al.* Integration of MODIS land and atmosphere products with a coupled-process model to estimate gross primary productivity and evapotranspiration from 1 km to global scales. *Global Biogeochem. Cycles* 25, GB4017, https://doi.org/10.1029/2011GB004053 (2011).
32. Bonan, G. B. *et al.* Improving canopy processes in the Community Land Model version 4 (CLM4) using global flux fields empirically inferred from FLUXNET data. *J. Geophys. Res.* 116, G02014, https://doi.org/10.1029/2010JG001593 (2011).
33. Beer, C. *et al.* Terrestrial gross carbon dioxide uptake: global distribution and covariation with climate. *Science* 329, 834–838, doi: 10.1126/science.1184984 (2010).

34. Jung, M. *et al.* Global patterns of land-atmosphere fluxes of carbon dioxide, latent heat, and sensible heat derived from eddy covariance, satellite, and meteorological observations. *J. Geophys. Res.-Biogeosciences* 116, G00J07, https://doi.org/10.1029/2010JG001566 (2011).

35. Badgley, G., Anderegg, L. D. L., Berry, J. A. & Field, C. B. Terrestrial Gross Primary Production: Using NIR V to Scale from Site to Globe. *Glob. Chang. Biol.* 0, 1-10 (2019). doi:10.1111/gcb.14729.

36. Joiner, J. *et al.* Estimation of Terrestrial Global Gross Primary Production (GPP) with Satellite Data-Driven Models and Eddy Covariance Flux Data. *Remote Sensing* 10, 1346, https://doi.org/10.3390/rs10091346 (2018).

37. Yuan, W. *et al.* Global estimates of evapotranspiration and gross primary production based on MODIS and global meteorology data. *Remote Sens. Environ.* 114, 1416–1431, https://doi.org/10.1016/j.rse.2010.01.022 (2010).

38. Zhang, Y. *et al.* A global moderate resolution dataset of gross primary production of vegetation for 2000-2016. *Sci. Data* 4, 170165, doi: 10.1038/sdata.2017.165 (2017).

39. Jiang, C. & Ryu, Y. Multi-scale evaluation of global gross primary productivity and evapotranspiration products derived from Breathing Earth System Simulator (BESS). *Remote Sens. Environ.* 186, 528–547, https://doi.org/10.1016/j.rse.2016.08.030 (2016).

40. Stocker, B. D. *et al.* Drought impacts on terrestrial primary production underestimated by satellite monitoring. *Nat. Geosci.* 12, 264–270, https://doi.org/10.1038/s41561-019-0318-6 (2019).

41. Chen, J. M. *et al.* Effects of foliage clumping on the estimation of global terrestrial gross primary productivity. *Global Biogeochem. Cycles* 26, GB1019, https://doi.org/10.1029/2010GB003996 (2012).

42. Ito, A. A global-scale simulation of the CO_2 exchange between the atmosphere and the terrestrial biosphere with a mechanistic model including stable carbon isotopes, 1953–1999. *Tellus B: Chem. Phys. Meteorol.* 55, 596-612, https://doi.org/10.1034/j.1600-0889.2003.01423.x (2003).

43. Zhao, M., Running, S. W., & Nemani, R. R. Sensitivity of Moderate Resolution Imaging Spectroradiometer (MODIS) terrestrial primary production to the accuracy of meteorological reanalyses. *J. Geophys. Res: Biogeosciences.* 111, 1-13, https://doi.org/10.1029/2004JG000004 (2006).

44. Demarty, J., Chevallier, F., Friend, A. D., Viovy, N., Piao, S., & Ciais, P. Assimilation of global MODIS leaf area index retrievals within a terrestrial biosphere model. Geophys. Res. Lett. 34, L15042, https://doi.org/10.1029/2007GL030014 (2007).

45. Ryu, Y., Berry, J. A., & Baldocchi, D. D. What is global photosynthesis? History, uncertainties and opportunities. *Remote Sens. Environ.* 223, 95-114, https://doi.org/10.1016/j.rse.2019.01.016 (2019).

46. Jian, J., Gough, C., Sihi, D., Hopple, A. M. & Bond-Lamberty, B. Collar Properties and Measurement Time Confer Minimal Bias Overall on Annual Soil Respiration Estimates in a Global Database. *Journal of Geophysical Research: Biogeosciences* 125, e2020JG006066, https://doi.org/10.1029/2020JG006066 (2020).

47. Barba, J. *et al.* Comparing ecosystem and soil respiration: Review and key challenges of tower-based and soil measurements. *Agric. For. Meteorol.* 249, 434–443, https://doi.org/10.1016/j.agrformet.2017.10.028 (2018).

48. Sánchez-Cañete, E. P., Barron-Gafford, G. A., & Chorover, J. A considerable fraction of soil-respired CO_2 is not Emitted Directly to the Atmosphere. *Scientific Reports* 8, 1, 13518, https://doi.org/10.1038/s41598-018-29803-x (2018).

49. Zhang, Y., Joiner, J., Gentine, P. & Zhou, S. Reduced solar-induced chlorophyll fluorescence from GOME-2 during Amazon drought caused by dataset artifacts. *Glob. chang. Biol.* 24, 2229–2230, https://doi.org/10.1111/gcb.14134 (2018).

50. Lyapustin, A. *et al.* Scientific impact of MODIS C5 calibration degradation and C6+ improvements. *Atmos. Meas. Tech.* 7, 4353-4365, https://doi.org/10.5194/amt-7-4353-2014 (2014).

51. Karlsen, S. R., Anderson, H. B., van der Wal, R. & Hansen, B. B. A new NDVI measure that overcomes data sparsity in cloud-covered regions predicts annual variation in ground-based estimates of high arctic plant productivity. *Environ. Res. Lett.* 13, 025011, https://doi.org/10.1088/1748-9326/aa9f75 (2018).

52. Prince, S. D. & Goward, S. N. Global Primary Production: A Remote Sensing Approach. *J. Biogeogr.* 22, 815–835, https://doi.org/10.2307/2845983 (1995).

53. Myers-Smith, I. H. *et al.* Complexity revealed in the greening of the Arctic. *Nat. Clim. Chang.* 10, 106–117, https://doi.org/10.1038/s41558-019-0688-1 (2020).

54. Huete, A. R. & Jackson, R. D. Soil and atmosphere influences on the spectra of partial canopies. *Remote Sens. Environ.* 25, 89–105, https://doi.org/10.1016/0034-4257(88)90043-0 (1988).
55. Keenan, T. F. *et al.* Widespread inhibition of daytime ecosystem respiration. *Nat. Ecol. Evol.* 33, 407-415, doi: 10.1038/s41559-019-0809-2 (2019).
56. Ito, A. A historical meta-analysis of global terrestrial net primary productivity: Are estimates converging? *Glob. Chang. Biol.* 17, 3161–3175, https://doi.org/10.1111/j.1365-2486.2011.02450.x (2011).
57. Doughty, C. E. & Field, C. B. Agricultural net primary production in relation to that liberated by the extinction of Pleistocene mega-herbivores: an estimate of agricultural carrying capacity? *Environ. Res. Lett.* 5, 044001, doi:10.1088/1748-9326/5/4/044001 (2010).
58. Whittaker, R. H. & Likens, G. E. Carbon in the biota. *Brookhaven Symp. Biol.* 281–302 (1973).
59. Werf, G. R. van der *et al.* Global fire emissions estimates during 1997–2016. *Earth Syst. Sci. Data* 9, 697–720, https://doi.org/10.5194/essd-9-697-2017 (2017).
60. Crutzen, P. J. & Andreae, M. O. Biomass burning in the tropics: impact on atmospheric chemistry and biogeochemical cycles. *Science* 250, 1669–1678, DOI: 10.1126/science.250.4988.1669 (1990).
61. Gerber, S., Joos, F. & Prentice, I. C. Sensitivity of a dynamic global vegetation model to climate and atmospheric CO_2. *Glob. Chang. Biol.* 10, 1223–1239, https://doi.org/10.1111/j.1529-8817.2003.00807.x (2004).
62. Piao, S. *et al.* Spatiotemporal patterns of terrestrial carbon cycle during the 20th century. *Global Biogeochem. Cycles* 23, GB4026, https://doi.org/10.1029/2008GB003339 (2009).
63. Zaehle, S., Sitch, S., Smith, B. & Hatterman, F. Effects of parameter uncertainties on the modeling of terrestrial biosphere dynamics. *Global Biogeochem. Cycles* 19, GB3020, https://doi.org/10.1029/2004GB002395 (2005).
64. Mieville, A. *et al.* Emissions of gases and particles from biomass burning during the 20th century using satellite data and an historical reconstruction. *Atmos. Environ.* 44, 1469–1477, https://doi.org/10.1016/j.atmosenv.2010.01.011 (2010).
65. Mouillot, F., Narasimha, A., Balkanski, Y., Lamarque, J.-F. & Field, C. B. Global carbon

emissions from biomass burning in the 20th century. *Geophys. Res. Lett.* 33, L01801, https://doi.org/10.1029/2005GL024707 (2006).

66. Schultz, M. G. *et al.* Global wildland fire emissions from 1960 to 2000. *Global Biogeochem. Cycles* 22, GB2002, https://doi.org/10.1029/2007GB003031 (2008).
67. Le Quéré, C. *et al.* Global Carbon Budget 2015. *Earth Syst. Sci. Data* 7, 349–396, https://doi.org/10.5194/essd-7-349-2015 (2015).
68. Cole, J. J. *et al.* Plumbing the Global Carbon Cycle: Integrating Inland Waters into the Terrestrial Carbon Budget. *Ecosystems* 10, 172–185, doi: 10.1007/s10021-006-9013-8 (2007).
69. Tranvik, L. J. *et al.* Lakes and reservoirs as regulators of carbon cycling and climate. *Limnol. Oceanogr.* 54, 2298–2314, https://doi.org/10.4319/lo.2009.54.6_part_2.2298 (2009).
70. Deemer, B. R. *et al.* Greenhouse Gas Emissions from Reservoir Water Surfaces: A New Global Synthesis. *Bioscience* 66, 949–964, https://doi.org/10.1093/biosci/biw117 (2016).
71. Raymond, P. A. *et al.* Global carbon dioxide emissions from inland waters. *Nature* 503, 355–359, doi:10.1038/nature12760 (2013).
72. Guenther, A. B. *et al.* The Model of Emissions of Gases and Aerosols from Nature version 2.1 (MEGAN2.1): an extended and updated framework for modeling biogenic emissions. *Geosci. Model Dev.* 5, 1471–1492, https://doi.org/10.5194/gmd-5-1471-2012 (2012).
73. Ryan, M. G., Lavigne, M. B. & Gower, S. T. Annual carbon cost of autotrophic respiration in boreal forest ecosystems in relation to species and climate. *J. Geophys. Res.-Atmospheres* 102, 28871–28883, https://doi.org/10.1029/97JD01236 (1997).
74. Allen, L. H., Jr. & Lemon, E. R. Carbon dioxide exchange and turbulence in a Costa Rican tropical rain forest. *Vegetation and the Atmosphere* (Academic Press, London, 1976).
75. Yoda, K. Community respiration in a lowland rain forest in Pasoh, Peninsular Malaysia. *Jap. J. Ecol.* 33, 183–197, https://doi.org/10.18960/seitai.33.2_183 (1983).
76. Yoda, K. Estimation of community respiration. *Biological production in a warm-temperate evergreen oak forest of Japan, JIBP Synthesis* 18, 112–131 (1978).
77. Edwards, N. T. *et al.* Carbon metabolism in terrestrial ecosystems. *Dynamic Properties of Forest Ecosystems. 499–536* (Cambridge University Press, Cambridge, 1981).
78. Ryan, M. G., Hubbard, R. M., Pongracic, S., Raison, R. J. & McMurtrie, R. E. Foliage, fine-

root, woody-tissue and stand respiration in Pinus radiata in relation to nitrogen status. *Tree Physiol.* 16, 333–343, https://doi.org/10.1093/treephys/16.3.333 (1996).

79. Law, B. E., Ryan, M. G. & Anthoni, P. M. Seasonal and annual respiration of a ponderosa pine ecosystem. *Glob. Chang. Biol.* 5, 169–182, https://doi.org/10.1046/j.1365-2486.1999.00214.x (1999).
80. Suleau, M. *et al.* Respiration of three Belgian crops: Partitioning of total ecosystem respiration in its heterotrophic, above- and below-ground autotrophic components. *Agric. For. Meteorol.* 151, 633–643, https://doi.org/10.1016/j.agrformet.2011.01.012 (2011).
81. Granier, A. *et al.* The carbon balance of a young Beech forest. *Funct. Ecol.* 14, 312–325, https://doi.org/10.1046/j.1365-2435.2000.00434.x (2000).
82. Chen, X., Hutley, L. B. & Eamus, D. Carbon balance of a tropical savanna of northern Australia. *Oecologia* 137, 405–416, doi:10.1007/s00442-003-1358-5 (2003).
83. Bolstad, P. V., Davis, K. J., Martin, J., Cook, B. D. & Wang, W. Component and whole-system respiration fluxes in northern deciduous systems. *Tree Physiol.* 24, 493–504, https://doi.org/10.1093/treephys/24.5.493 (2004).
84. Curtis, P. S. *et al.* Respiratory carbon losses and the carbon-use efficiency of a northern hardwood forest, 1999–2003. *New Phytol.* 167, 437–456, https://doi.org/10.1111/j.1469-8137.2005.01438.x (2005).
85. Davidson, E. A., Richardson, A. D., Savage, K. E. & Hollinger, D. Y. A distinct seasonal pattern of the ratio of soil respiration to total ecosystem respiration in a spruce-dominated forest. *Glob. Chang. Biol.* 12, 230–239, https://doi.org/10.1111/j.1365-2486.2005.01062.x (2006).
86. Nagy, M. T., Janssens, I. A., Curiel Yuste, J., Carrara, A. & Ceulemans, R. Footprint-adjusted net ecosystem CO2 exchange and carbon balance components of a temperate forest. *Agric. For. Meteorol.* 139, 344–360, https://doi.org/10.1016/j.agrformet.2006.08.012 (2006).
87. Zhang, Y. *et al.* Annual variation of carbon flux and impact factors in the tropical seasonal rain forest of xishuangbanna, SW China. *Sci. China Ser. D Earth Sci.* 49, 150–162, doi: 10.1007/s11430-006-8150-4 (2006).
88. Jassal, R. S. *et al.* Components of ecosystem respiration and an estimate of net primary productivity of an intermediate-aged Douglas-fir stand. *Agric. For. Meteorol.* 144, 44–57,

https://doi.org/10.1016/j.agrformet.2007.01.011 (2007).

89. Zha, T. *et al.* Total and component carbon fluxes of a Scots pine ecosystem from chamber measurements and eddy covariance. *Ann. Bot.* 99, 345–353, https://doi.org/10.1093/aob/mcl266 (2007).

90. Keith, H. *et al.* Multiple measurements constrain estimates of net carbon exchange by a Eucalyptus forest. *Agric. For. Meteorol.* 149, 535–558, https://doi.org/10.1016/j.agrformet.2008.10.002 (2009).

91. Kolari, P. *et al.* CO2 exchange and component CO2 fluxes of a boreal Scots pine forest. *Boreal Environ. Res.* 14, 761-783 (2009).

92. Malhi, Y. *et al.* Comprehensive assessment of carbon productivity, allocation and storage in three Amazonian forests. *Glob. Chang. Biol.* 15, 1255–1274, https://doi.org/10.1111/j.1365-2486.2008.01780.x (2009).

93. Wieser, G. *et al.* Respiratory fluxes in a Canary Islands pine forest. *Tree Physiol.* 29, 457–466, https://doi.org/10.1093/treephys/tpp008 (2009).

94. Zhang, P., Tang, Y., Hirota, M., Yamamoto, A. & Mariko, S. Use of a regression method to partition sources of ecosystem respiration in an alpine meadow. *Soil Biol. Biochem.* 41, 663–670, https://doi.org/10.1016/j.soilbio.2008.12.026 (2009).

95. Hermle, S., Lavigne, M. B., Bernier, P. Y., Bergeron, O. & Paré, D. Component respiration, ecosystem respiration and net primary production of a mature black spruce forest in northern Quebec. *Tree Physiol.* 30, 527–540, https://doi.org/10.1093/treephys/tpq002 (2010).

96. Ryan, M. G. *et al.* Factors controlling Eucalyptus productivity: How water availability and stand structure alter production and carbon allocation. *For. Ecol. Manage.* 259, 1695–1703, https://doi.org/10.1016/j.foreco.2010.01.013 (2010).

97. Tan, Z. *et al.* Carbon balance of a primary tropical seasonal rain forest. *J. Geophys. Res.* 115, 921, https://doi.org/10.1029/2009JD012913 (2010).

98. Jans, W. W. P. *et al.* Carbon exchange of a maize (Zea mays L.) crop: Influence of phenology. *Agric. Ecosyst. Environ.* 139, 316–324, https://doi.org/10.1016/j.agee.2010.06.008 (2010).

99. Campoe, O. C., Stape, J. L., Laclau, J.-P., Marsden, C. & Nouvellon, Y. Stand-level patterns of carbon fluxes and partitioning in a Eucalyptus grandis plantation across a

gradient of productivity, in São Paulo State, Brazil. *Tree Physiol.* 32, 696–706, https://doi.org/10.1093/treephys/tps038 (2012).

100. Matteucci, M., Gruening, C., Goded Ballarin, I., Seufert, G. & Cescatti, A. Components, drivers and temporal dynamics of ecosystem respiration in a Mediterranean pine forest. *Soil Biol. Biochem.* 88, 224–235, https://doi.org/10.1016/j.soilbio.2015.05.017 (2015).
101. Bond-Lamberty, B., Wang, C. & Gower, S. T. A global relationship between the heterotrophic and autotrophic components of soil respiration? *Glob. Chang. Biol.* 10, 1756–1766, https://doi.org/10.1111/j.1365-2486.2004.00816.x (2004).
102. Friedl, M. A. *et al.* Global land cover mapping from MODIS: algorithms and early results. *Remote Sens. Environ.* 83, 287–302, https://doi.org/10.1016/S0034-4257(02)00078-0 (2002).
103. Fick, S. E. & Hijmans, R. J. WorldClim 2: new 1-km spatial resolution climate surfaces for global land areas. *Int. J. Climatol.* 37, 4302-4315, https://doi.org/10.1002/joc.5086 (2017).
104. Lamarque, J. F., Dentener, F. & McConnell, J. Multi-model mean nitrogen and sulfur deposition from the Atmospheric Chemistry and Climate Model Intercomparison Project (ACCMIP): evaluation of historical and projected future changes. *Atmospheric* 13, 7997-8018, https://doi.org/10.5194/acp-13-7997-2013 (2013).
105. Hengl, T. *et al.* SoilGrids250m: Global gridded soil information based on machine learning. *PLoS One* 12, e0169748, https://doi.org/10.1371/journal.pone.0169748 (2017).
106. Spawn, S. A. & Gibbs, H. K. Global Aboveground and Belowground Biomass Carbon Density Maps for the Year 2010. *ORNL DAAC.* https://doi.org/10.3334/ORNLDAAC/1763 (2020).
107. Huete, A. *et al.* Overview of the radiometric and biophysical performance of the MODIS vegetation indices. *Remote Sens. Environ.* 83, 195–213, https://doi.org/10.1016/S0034-4257(02)00096-2 (2002).
